# Reproduction of overall spontaneous pain pattern by manual stimulation of active myofascial trigger points in fibromyalgia patients

**DOI:** 10.1186/ar3289

**Published:** 2011-03-22

**Authors:** Hong-You Ge, Ying Wang, César Fernández-de-las-Peñas, Thomas Graven-Nielsen, Bente Danneskiold-Samsøe, Lars Arendt-Nielsen

**Affiliations:** 1 Department of Health Science and Technology, Aalborg University, Fredrik Bajersvej 7, Aalborg, DK-9220, Denmark; 2 The Parker Institute, Frederiksberg Hospital, Ndr. Fasanvej 57, Frederiksberg, DK-2000, Denmark; 3 Department of Physical Therapy, Occupational Therapy, Rehabilitation and Physical Medicine, Universidad Rey Juan Carlos, Avda. de Atenas, s/n. Alcorcón, Madrid, 28922, Spain

## Abstract

**Introduction:**

It has previously been reported that local and referred pain from active myofascial trigger points (MTPs) in the neck and shoulder region contribute to fibromyalgia (FM) pain and that the pain pattern induced from active MTPs can reproduce parts of the spontaneous clinical FM pain pattern. The current study investigated whether the overall spontaneous FM pain pattern can be reproduced by local and referred pain from active MTPs located in different muscles.

**Methods:**

A spontaneous pain pattern in FM was recorded in 30 FM patients and 30 healthy subjects served as controls. Local and referred pain patterns induced from active (patients) and latent (controls) MTPs were recorded following manual stimulation. The existence of MTPs was confirmed by intramuscular electromyographical registration of spontaneous electrical activity.

**Results:**

Local and referred pain areas induced from key active MTPs in FM were larger than pain areas from latent MTPs in healthy controls (*P *< 0.001), but were similar to the overall spontaneous FM pain area in FM (*P *> 0.05). The induced pain area was positively associated with current spontaneous pain intensity in FM (*P *< 0.01). The locations of key active MTPs in FM patients were found to have latent MTPs in healthy subjects. The muscles containing key active MTPs in FM are often observed in the muscles of extensor digitorum, trapezius, infraspinatus in the upper part of the body and of quadratus lumborum, gluteus medius in the lower part of the body.

**Conclusions:**

The overall spontaneous FM pain pattern can be reproduced by mechanical stimulation of active MTPs located in different muscles, suggesting that fibromyalgia pain is largely composed of pain arising from muscle pain and spasm. Targeting active MTPs and related perpetuating factors may be an important strategy in FM pain control.

**Trial registration:**

ISRCTN ISRCTN43167547.

## Introduction

A defining characteristic for fibromyalgia (FM) is chronic widespread pain of musculoskeletal origin [1]. The widespread pain, which constitutes the overall spontaneous pain pattern in FM, is not uniformly distributed all over the body [2]. Increasing evidence suggests that nociceptive inputs from muscle tissues and myofascial trigger points (MTPs) in particular are important in the initiation and/or maintenance of FM pain and central sensitization. An anesthetic epidural blockade with lidocaine completely eliminates spontaneous pain and tender points and/or myofascial trigger points (MTPs) in FM patients [3], suggesting the importance of peripheral nociceptive inputs in FM. Accordingly, a single intramuscular anesthetic injection into the mid-point of the upper trapezius muscle, a typical site of active MTP in FM, significantly increases local pain thresholds and decreases remote secondary heat hyperalgesia in FM patients [4] and injections of anesthetic into multiple MTPs reverse mechanical hyperalgesia and decrease overall pain intensity in patients with whiplash and FM [5,6]. Manual provocation of active MTPs reproduced substantial parts of the clinical pain pattern experienced by FM patients [7,8]. These evidences suggest that active MTPs contribute significantly to the overall spontaneous pain pattern in FM. MTPs may occur in any skeletal muscle in multiple or single locations [9]. In FM patients, active MTPs may be observed in different muscles [10] and at multiple locations in a single muscle [7], apart from active MTPs in predetermined tender point sites in FM [8,10]. However, it is unknown whether stimulation of active MTPs in different muscles can reproduce the overall spontaneous pain pattern in FM.

The aim of the current study is to assess whether the overall spontaneous FM pain pattern can be reproduced by the local and referred patterns from multiple active MTPs in different muscles.

## Materials and methods

### Participants

The sample consisted of 30 women with fibromyalgia (FM group, mean age: 53.6 ± 2.5 yr; mean weight: 68.2 ± 3.5 kg; mean height: 173 ± 29.8 cm) and 30 age- and gender-matched healthy controls (control group, mean age: 52.6 ± 2.5 yr; mean weight: 65.5 ± 3.1 kg; mean height: 171 ± 32.9 cm). Only women between the ages of 18 and 70 were recruited for the study. Disease duration of FM was 10.5 ± 0.7 yr. There were no significant differences in age, weight, and height between these two groups. This study was approved by the local Ethics Committee (VN 20080018) and conducted in accordance with the Helsinki Declaration. Informed consent was obtained from all subjects. The participants were recruited through a local FM support group and through rheumatology clinics.

The patients had their FM diagnosis confirmed by a physician according to The American College of Rheumatology 1990 criteria for the classification of fibromyalgia [1], had an average pain rating of 6.2 ± 2.2 cm for the past 24 hours and of 5. 3 ± 2.2 cm for the current spontaneous pain on the day of experiment on a 0 to 10 cm visual analogue scale (VAS, 0 = no pain and 10 = worst pain imaginable). Out of 30 FM patients, 26 patients were taking one or more kind(s) of the following medications: non-steroidal anti-inflammatory drugs (16/20), opioid products (8/20), sedatives (8/20), antidepressants (4/20), and gabapentinoids (6/20). The FM patients were not excluded if depressed or taking antidepressant medications and/or analgesics.

The control group had no current spontaneous pain, no major pain experience during the past month prior to the experiment, and no pain-related diagnoses (for example, FM, osteoarthritis, rheumatoid arthritis, low back pain, and so on).

### Experimental protocol

Each subject was asked to rate the current overall spontaneous pain intensity and to draw on an anatomical map the pain areas felt on the day of the experiment. These data were open to the first experimenter who was responsible for the identification of active MTPs in FM patients and latent MTPs in healthy subjects.

The first experimenter identified key active, but not latent, MTPs in different muscles with the aim to reproduce each patient's pain pattern. However, only one key active MTP was identified in one muscle to minimize the suffering of the patients. A key active MTP is defined in the current study as the most tender spot together with the largest nodule compared to other spots when flat palpation is applied. The evoked local and referred pain pattern from each key active MTP was recorded. These key active MTPs were marked with visible ink and the locations of these key active MTPs were noted. The locations of these key active MTPs of a FM patient were then mirrored onto an age-matched healthy subject and served as locations for examination of latent MTPs. The second experimenter was responsible for the intramuscular EMG examination of MTPs at marked points searching for the spontaneous electrical activity in FM and healthy subjects and was blinded to other data on the subjects.

### Manual identification of active MTPs in FM patients and latent MTPs in healthy subjects

Manual identification of MTPs in different muscles was done by snapping palpation (first to locate a taut band of muscle and place the fingertip at right angles, and then moving the thumb tip back and forth to roll the underlying fibers) to induce local twitch response, and flat palpation (using the padded aspect of the thumb at a right angle to the muscle fibers and applying pressure against the underlying tissue or bone) to induce local pain and referred pain. The applied pressure to each point was about 4 kg and lasted for 10 sec. The potential locations of active MTPs in FM patients were determined according to the muscle specific local and referred pain patterns described in The Trigger Point Manual [11]. The presence of an MTP was determined according to proposed diagnostic criteria of MTPs [11,12]: an active MTP has to meet item 4) and two of the first three items: 1) presence of a palpable taut band, 2) a tender spot within a taut band, 3) local twitch response by snapping palpation of the taut band, and 4) referred pain evoked by flat palpation of the tender spot which reproduces the patient's complaints. A latent MTP has to meet two of the first three items in the criteria for an active MTP, and/or referred pain evoked by flat palpation of the tender spot, which does not reproduce the subject's complaints. In order to determine if local and referred pain reproduce the patient's complaints, the examiner would ask, "Do you feel any change in sensation to any area?" If the subject replied in the affirmative, the examiner asked, "Is the feeling (or pain) just like the one that is a problem to you?" as used in previous studies [7,13]. The local and referred pain pattern 10 sec following flat palpation on each point was drawn by the subjects on an anatomical map and later digitized (ACECAD D9000+, ACE CAD Enterprise Co., Ltd., Hsin Tien City, Taiwan, ROC) and expressed as an arbitrary unit for further analysis.

### Intramuscular electromyographic (EMG) examination of MTPs

EMG registration of spontaneous electrical activity (SEA) is the only electrophysiological method to document the existence of an MTP. In the current study, EMG registration of SEA was used to confirm or refute the existence of an MTP following manual identification as shown previously [8]. The amplitude of SEA larger than 50 uV was considered a threshold parameter for confirmation of an MTP. The area of skin at the marked location was cleaned with isopropyl alcohol. One pair of bipolar surface EMG electrodes (Neuroline 720-01-k, Ølstykke, Denmark, intra-electrode distance of 2 cm) was placed 2 cm away from each MTP site following skin preparation. The surface electrodes were used to ensure that the muscle under investigation was relaxed prior to needle EMG examination. During the EMG needle insertion, a thumb palpated the taut band and located the most tender spot on a taut band and applied slightly downward pressure just enough to fix the underlying tissue in place. The needle insertion was redirected twice if the first insertion failed to find the SEA. The purpose of intramuscular EMG examination was to find the existence of SEA, not aimed at finding a point with the highest amplitude of SEA. A longer intramuscular EMG needle electrode (Neuroline concentric, 75 × 0.65 mm (3" × 23 G)) was used to register SEA in deep or thick muscles, such as the gluteus medius and/or quadratus lumborum. A shorter intramuscular EMG needle electrode (Neuroline concentric, 25 × 0.45 mm (1" × 26 G)) was used to register SEA in superficial or thin muscles, such as the upper trapezius and/or limb muscles. The referred pain and local twitch response following needle insertion were not recorded. When a resting SEA from an MTP was shown on the EMG monitor during EMG needle insertion, the EMG signals from surface and intramuscular electrodes were then recorded for 4 s. Standard filter settings (5 Hz-1 kHz), gain (100 uV/div), and sweep speed (20 ms/div) were used on the electromyography system (Keypoint, Dantec Medical, Skovlunde, Denmark). EMG signals were sampled at 2 kHz and stored for offline inspection.

### Statistics

A two-way analysis of variance (ANOVA) was used to compare the differences in local and referred pain areas between patient and control groups. If significant, *post-hoc *pair-wise multiple comparisons were performed by Student-Newman-Keuls Method. Pearson product moment correlation was used to estimate the correlation coefficient between overall spontaneous pain intensity and the MTP evoked pain area in FM. The Chi-square test was used to 1) compare the difference in the positive rate of manual palpation and intramuscular EMG identification of a latent MTP in healthy subjects and an key active MTP in FM. Values in the text and figures are expressed as mean ± standard error (SEM) of the mean. Significance level was set at *P *< 0.05.

## Results

### Overall spontaneous FM pain and MTP-evoked pain

**Figure 1:**
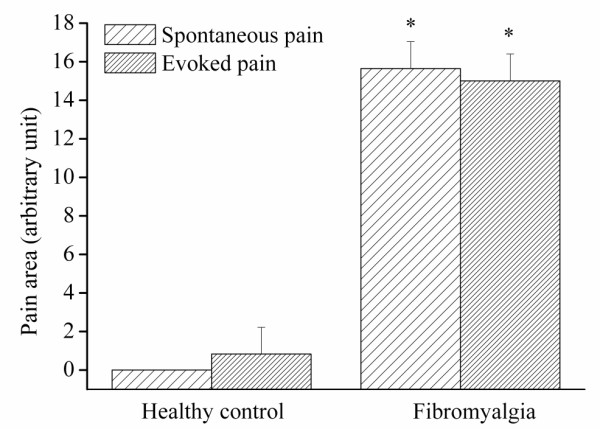
**Spontaneous and evoked pain areas**. Evoked local and referred pain area from myofascial trigger points in fibromyalgiaand healthy controls. Evoked pain area and spontaneous pain area are significantly larger in FM than controls (both, *P *< 0.001). Note: no spontaneous pain in healthy controls.

**Figure 2:**
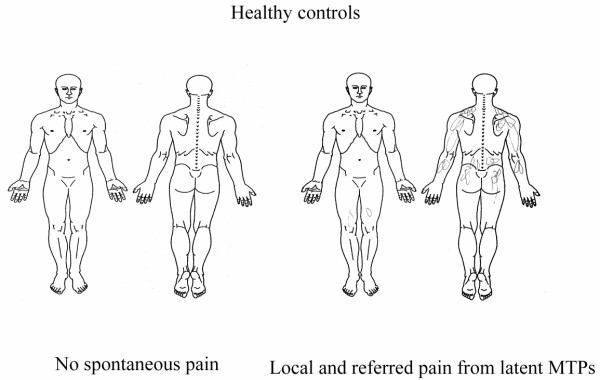
**Spontaneous and evoked pain in healthy controls**. Local and referred pain pattern from latent myofascial trigger points (MTPs) in healthy controls.

**Figure 3:**
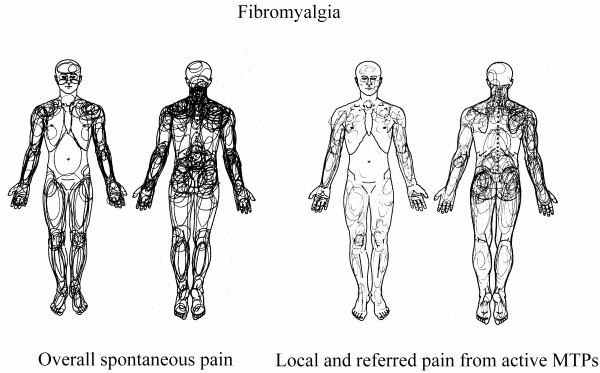
**Spontaneous and evoked pain in FM patients**. The overall spontaneous pain pattern and the local and referred pain pattern from key active myofascial trigger points (MTPs) in FM. Note: the local and referred pain pattern was lightly shaded in purpose to denote the induced pain from key active MTPs.

A two-way ANOVA revealed a significant difference in spontaneous FM pain and evoked pain areas between FM and controls groups (F = 114.4, *P *< 0.001, Figure 1), but no significant differences were observed in the area between spontaneous pain and MTP-evoked pain within the FM group and control group (F = 0.0044, *P *= 0.947, Figure 1). In healthy subjects where no spontaneous pain was reported, local and referred pain were evoked from latent MTPs (Figure 2). In FM patients, the local and referred pain patterns evoked from key active MTPs were similar to the overall spontaneous pain pattern (Figure 3).

**Figure 4:**
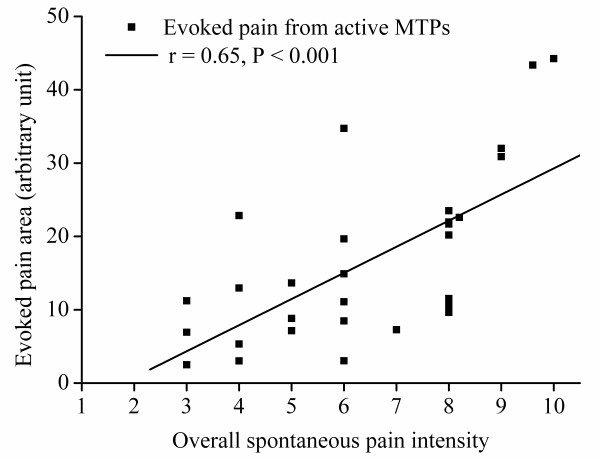
**Correlation between spontaneous pain and evoked pain in FM**. Correlation between induced pain areas from key active myofascial trigger points (MTPs) with overall spontaneous pain intensity in FM.

There was a significant positive correlation between the evoked pain area from all key active MTPs and the overall spontaneous pain intensity in FM (r = 0.65, *P *= 0.0001, Figure 4).

### Muscles with key active MTPs in FM and with latent MTPs in healthy controls

In the current study, the locations for the examination of latent MTPs in healthy subjects were mirrored from the locations of key active MTPs in FM.

The first three muscles in the upper part of the body in order of occurrence of key active MTPs in FM confirmed with intramuscular EMG are: Extensor Digitorum, Upper Trapezius, and the Supraspinatus. The first three muscles in the lower part of the body in order of occurrence of key active MTPs confirmed with intramuscular EMG are: quadratus lumborum, gluteus medius, and vastus medialis (Table 1). The key active MTPs in other muscles contributed to the overall spontaneous pain pattern are listed in Table 1.

**Table 1:** The number of key active MTPs in fibromyalgia (N = 30)

Muscles	Left side	Right side	Subtotal
Subocciput	4	5	9
Temporalis (*TrP3*)	1	1	2
Upper trapezius (*TrP2*)	20	21	41
Supraspinatus (*mid) *)	15	14	30
Infraspinatus (*mid*)	12	13	25
Pectoris major (*mid*)	5	7	12
Posterior deltoid (*mid*)	6	8	14
Extensor digitorum (2 cm distal to lateral epicondyle)	22	23	43
Rhomboid major (*mid*)	2	3	5
Triceps (*mid*)	2	3	5
Biceps (*mid*)	2	2	4
Quadratus lumborum (tip of 3^rd ^transverse process)	20	23	43
Gluteus medius (*mid*)	12	13	25
Vastus medialis (*TrP1*)	9	8	18
Rectus femoris (*proximal attachment*)	4	5	9
Tibialis anterior (*proximal attachment*)	2	3	5
Peroneous longus (*mid*)	2	2	4
Rectus abdominis (*2 cm lateral to umbilicus*)	1	1	2
Gastrocnemius (*TrP1,2*)	2	2	4
Plantar muscles (*mid*)	2	3	5
Subtotal	146	159	305

MTPs, myofascial trigger points confirmed by intramuscular EMG examination.

The italicized texts in the brackets indicate the actual locations of key MTPs. Mid, mid portion of the muscle. TrP1 or TrP2 or TrP3: trigger point locations defined in Travell and Simons book: *Myofascial Pain and Dysfunction the Trigger Point Manual *[11].

A total of 308 latent MTPs were identified in 30 healthy subjects with manual palpation, of which 304 MTPs were confirmed by intramuscular EMG examination. In 30 FM patients, a total of 308 key active MTPs were identified with manual palpation, of which 305 were confirmed by intramuscular EMG examination. The occurrence of latent MTPs in healthy subjects in corresponding locations to FM was not significantly different to the occurrence of key active MTPs in FM (χ^2 ^= 0.14, *P *= 0.70).

## Discussion

The main finding of the current study is the reproduction of patient-specific overall spontaneous pain pattern in FM by manual stimulation of key active MTPs. This is the first study to show that the overall spontaneous pain pattern in each FM patient can be decomposed into muscle-specific local and referred pain patterns from active MTPs. A significantly positive correlation was found between evoked pain area and current spontaneous pain intensity in FM. The current study supports the notion that active MTPs are the major peripheral pain generator in FM.

### Reproduction of overall spontaneous pain pattern from active MTPs

There were large between-subject differences in real-time FM pain reports and the fluctuation in pain report was constant over time within individuals [14]; thus in the current study, we identified key active MTPs in FM according to the overall spontaneous pain pattern on the day of experiment. The results show that overall local and referred pain areas and pain patterns induced from key active MTPs are similar to those of the overall spontaneous pain in FM. Further, the local and referred pain area from active MTPs in FM is also positively related to current overall spontaneous pain intensity in the current study. These results suggest that active MTPs are the major peripheral pain generators in FM [15]. The current results are consistent with previous findings showing that the predetermined tender point sites in FM [1] are MTPs, either active or latent [8,10] and that local and referred pain from multiple active MTPs contribute to FM pain in the neck and shoulder region [7]. Active MTPs have greater potential than latent MTPs to induce central sensitization as evidenced by a larger local and referred pain area in FM than that from latent MTPs in healthy controls. Decreasing the peripheral input from a tender point site (an MTP) in FM significantly decreases the secondary heat hyperalgesia [4]. Further, consecutive anesthetic injections into active MTPs in FM significantly decreases spontaneous pain intensity associated with an increased mechanical pain threshold at predetermined tender point sites [6]. Thus, it is quite obvious that active MTPs can serve as potent ongoing peripheral nociceptive inputs initiating and maintaining central sensitization in FM.

Understanding the nature of FM pain may provide significant clues to clinical management of FM. The results in the current study show the widespread spontaneous pain pattern in FMS is a summation of multiple regional pains due to active MTPs. A regional pain in FMS is from local active MTPs and/or referred from remote active MTPs. A previous study shows that the overall spontaneous FMS pain is not diffuse body pain but is located in certain body areas [2], as also depicted by pain drawing of spontaneous pain pattern by FM patients in a recent study [8] and the current study. Targeting active MTPs may significantly improve FM pain and dysfunction. A recent study targeting active MTPs in FM has shown encouraging results in decreasing spontaneous FM pain and mechanical hyperalgesia in FM [6]. In addition to the peripheral sensitization by active MTPs, central sensitization may also increase MTP sensitivity [16,17] and play a significant role in enhancing peripheral sensitization and normally nonpainful stimuli may be perceived as painful in FM [18].

### Muscles commonly harboring active MTPs in FM

From the overall spontaneous pain pattern observed in the current study, FM pain is heavily concentrated in the neck-shoulder-arm region and in the low back and gluteal region. Corresponding to the painful regions, more key active MTPs are identified in the muscles of extensor digitorum, upper trapezius, supraspinatus, and infraspinatus than other muscles in the neck-shoulder-arm region; more key active MTPs are identified in the muscles of quadratus lumborum and gluteus medias than other muscles in the low back and gluteal region. The quadratus lumborum muscle, which is not listed in the predetermined tender point sites in FM [1], especially needs attention when evaluating FM patients with low back pain. This key active MTP site is usually found around the tip of the third lumbar transverse process.

It is noteworthy that identification of key active MTPs should not be confined to the predetermined tender point sites in FM; there are other muscles harboring active MTPs. Thus, identification of active MTPs should follow the overall spontaneous pain pattern of each patient; that is, patient specific MTP identification strategy due to large between-subject differences in real-time FM pain reports [14].

It is also interesting to note that there was always found a latent MTP in healthy subjects at the corresponding locations of a key active MTP in FM in the current study. This may suggest that in this middle-aged healthy control group latent MTPs are very prevalent. Though there were no spontaneous pain reports in the healthy controls, they did report local and referred pain when some of the latent MTPs were manually stimulated. Thus, latent MTPs can be classified into latent MTPs with referred pain and without referred pain. Those latent MTPs with referred pain may underlie transient muscle pain episodes due to pressure or strain applied to the muscle in daily activities. The existence of a large number of latent MTPs in the healthy volunteers does not imply that most people have the potential for FM; rather activation of latent MTPs may underlie the development of local pain following acute and sustained muscle overload, psychophysical trauma, and other adverse events [10,11]. Only those with chronic local pain conditions for years may have the potential to develop FM [1,10].

There are limitations of the current study. First, the pain quality was not recorded following manual stimulation of active MTPs in FM. The dominant sensory abnormalities in FM are pressure pain, prickling, burning, and thermal hypersensitivity [19]. Large sample size studies are required to detail the characteristics of spontaneous pain and induced pain following mechanical stimulation of active MTPs in FM. Second, identification of all the locations of active MTPs in each muscle were not described in the current study, mapping of active MTPs in the muscles in, for example, the shoulder and low back regions may have important clinical significance.

## Conclusions

The overall spontaneous pain pattern in FM can be reproduced by active MTPs. Therapies targeted at active MTPs may significantly improve FM pain.

## Abbreviations

ANOVA: analysis of variance; EMG: electromyography; FM: fibromyalgia; MTPs: myofascial trigger points; SEA: spontaneous electrical activity; SEM: standard error of the mean; VAS: visual analogue scale.

## Competing interests

The authors declare that they have no competing interests.

## Authors' contributions

GHY and WY carried out data collection and analysis and drafted the manuscript. FC, GNT, DSB and LAN also participated in the design of the study and helped to draft the manuscript. All authors read and approved the final manuscript.
